# Replicative genomics can help *Helicobacter *fraternity usher in good times

**DOI:** 10.1186/1757-4749-2-25

**Published:** 2010-12-23

**Authors:** Niyaz Ahmed

**Affiliations:** 1 Pathogen Biology Laboratory, Department of Biotechnology, University of Hyderabad, Hyderabad, India; 2 Institute of Biological Sciences, University of Malaya, Kuala Lumpur, Malaysia

## Editorial

If we draw a parallel among two of the age-old scourges of humans, *Mycobacterium tuberculosis *and *Helicobacter pylori*, we will find a great superimposition of the two in terms of their history, ecology, geographic distribution and the fact that both the pathogens get fiercely engaged with host innate defenses and avoid clearance due to being highly adapted to their niches [1]. However, the pattern of their chromosomal evolution is very different [2]. Whole genome sequencing revealed that *M. tuberculosis *pangenome is of a closed type because of its largely being conserved [3] and that of *H. pylori *is an infinitely open one [4], meaning that dozens of new, undefined genes and genetic elements will be identified with the addition of each new genome to the existing pan-genome of the latter. This is good news for sequencing companies who were not happy with loosing the business opportunities with *Mycobacterium *research community but they can in a big way embark upon the *Helicobacter *sequencing assignments aimed at population genomics and towards finding new virulence factors and novel markers for strain identification [5].

The costs of whole genome sequencing have declined worldwide due to the feasibility and cost-effectiveness of the next generation sequencing platforms (such as Solexa). As these technologies permit multiplexing, bringing the costs further down, the *H. pylori *genome programs have almost become pedestrian. It is therefore a high time for even the lower resource settings to initiate genome inferred molecular epidemiology and switch over from genotyping to genome sequencing for strain identification. This has also a significant potential for the training of students who could pursue genome sequence annotation and comparative genomics projects for dissertations. This situation also has implications for countries and continents dealing with their 'enigmatic' position as to the *H. pylori *infection outcome - for example, the Indian enigma [6] or the Malaysian enigma [7].

*H. pylori*'s core genome is almost conserved with close to 1200 genes. However, its 'variome' comprised majorly of highly fluid, plasticity zones (PZs) could be of significant research interest because it contains novel genes and genetic elements whose composition and structure varies and evolves in association with the geographic descent of the strains and host ecology/physiology. Functional level understanding of such plastic genomic repertoires could be very significant in understanding mechanisms of adaptations and survival over time without being cleared by the innate immune system. Therefore, the wealth of comparative genomic data emanating from multiple whole genome sequencing projects will make it possible to systematically decipher functional consequences of genomic diversity at population level [5]. Such data will also be helpful to understand how genotypic information relevant in molecular epidemiology and evolutionary genetics could be scaled up (through functional screens of whole genome insertion, deletion and substitution patterns of single strains) to 'functional molecular infection epidemiology (FMIE). This approach could be highly useful in understanding of the interplay between pathogens and their hosts through descriptive host pathogen genomic variations encompassing some of the vital traits on both the sides such as adhesion, invasion, persistence and adaptation (bacterial side) as well as transient versus chronic infection, disease severity, progression to overt consequences and genetic susceptibility or resistance (host side).

Another important reason to pursue replicate genomics of *H. pylori *entails the need to study adaptation through evolution in a single host [8-11]. The nature and extent of genetic lesions that the chronically inhabiting *H. pylori *accumulates in a chronological manner during colonization of different host niches are not known; this needs in-depth analysis involving strains obtained hierarchically and sampled from different sites of individual stomachs to identify possible genetic exchanges or deletions occurring over time. Apart from this, geographically distinct strains and their multiple representatives could be sequenced to explore local advantages in terms of host adaptation or disease outcome; for example, *H. pylori *infection in the Indian population (despite a very high colonization rate of up to 90%) rarely leads to serious consequences [12] such as gastric cancer in a significant majority of patients who test positive for *H. pylori *infection (cancer rates <3.0 per 100000). Bacterial co-ordinates of such 'protection', if any, can be studied with the help of bacterial genome sequence data obtained from a number of strains.

Multiple whole genome sequencing of *H. pylori *has recently become an exciting regulatory tool in vaccine studies which advocate use of *H. pylori *as a vehicle for antigen delivery. The possibility of using short-term *H. pylori *colonisation of the stomach mucosa for antigen targeting is an attractive strategy for the development of an oral vaccine [13]. However, the inherent propensity of *H. pylori *to recombine and recast its genome needs to be monitored for any undesirable outcomes during the course of vaccination. Full genome sequencing could serve as a primary tool for validation of such vaccination approaches.

In conclusion, the *H. pylori *research community is witnessing good times amidst technology revolution. Genome data from high burden countries would add to the story in a more meaningful way. The data generated based on chronological and replicate genomics are likely to boost our understanding of the host-microbe interactions that could potentially occur with colonization by *H. pylori*, wherein, some could prove beneficial leading the co-evolved bacterium and its host to reach homeostasis akin to a 'symbiotic' relationship. Such descriptive host-pathogen associations, when systematically unraveled, would form the core of approaches heralding the era of FMIE.

## Competing interests

The author declares that they have no competing interests.

## Authors' contributions

NA conceived the idea of the subject editorial and drafted the manuscript.
